# Ccl2 deficiency protects against chronic renal injury in murine renovascular hypertension

**DOI:** 10.1038/s41598-018-26870-y

**Published:** 2018-06-05

**Authors:** Sonu Kashyap, Mazen Osman, Christopher M. Ferguson, Meryl C. Nath, Bhaskar Roy, Karen R. Lien, Karl A. Nath, Vesna D. Garovic, Lilach O. Lerman, Joseph P. Grande

**Affiliations:** 1 Department of Laboratory Medicine & Pathology, Mayo Clinic, Rochester, MN USA; 2 Division of Nephrology & Hypertension, Mayo Clinic, Rochester, MN USA; 3 Center for Regenerative Medicine, Mayo Clinic, Rochester, MN USA

## Abstract

Inflammation plays an important role in the pathogenesis of renal and cardiovascular disease in renovascular hypertension (RVH). Ccl2 is an important mediator of inflammation, and is induced within 24 hours following surgery to establish RVH in the murine 2 kidney 1 clip model, a time prior to onset of interstitial inflammation, fibrosis, or tubular atrophy. We tested the hypothesis that Ccl2 deficiency protects the stenotic kidney (STK) from development of chronic renal damage in mice with renovascular hypertension due to renal artery stenosis (RAS). RAS surgery was performed on wild type (WT) and Ccl2 knock out (KO) mice; animals were studied for four weeks. Renal blood flow was reduced to similar extent in both WT and Ccl2 KO mice with RVH. Perfusion of the stenotic kidney was significantly reduced in Ccl2 KO mice as assessed by magnetic resonance imaging (MRI). Stenotic kidney volume in WT, but not in Ccl2 KO mice, was significantly reduced following surgery. Cortical hypoxia was observed in the stenotic kidney of Ccl2 KO mice, as assessed by blood oxygen level-dependent MRI (BOLD-MRI). Ccl2 KO mice showed less cortical atrophy than WT RAS mice. Ccl2 deficiency reduced the number of infiltrating mononuclear cells and expression of *Ccl5*, *Ccl7*, *Ccl8*, *Ccr2* and *Cd20*6. We conclude that Ccl2 is a critical mediator of chronic renal injury in RVH.

## Introduction

Recent studies have linked both local and systemic inflammation in the pathogenesis of renal and cardiovascular disease in patients with renovascular hypertension (RVH)^1,2^. Indeed, systemic inflammation may be responsible for the significantly increased risk of mortality from cardiovascular disease in patients with chronic kidney disease (CKD)^3^. Chronic angiotensin II infusion promotes cardiac hypertrophy and remodeling where renal infiltration of macrophages (Mø) and T cells occurs as an early event^4–6^. In the two kidney 1 clip (2K1C) model of RVH, influx of inflammatory cells into the stenotic kidney occurs within one week and precedes the development of severe interstitial fibrosis and tubular atrophy^7–9^. Immunosuppressive therapy, at least in part, ameliorates renal damage in the angiotensin II infusion model^10^. These studies support the notion that therapies to target inflammation may have a role in the management of patients with RVH.

Of the many pro-inflammatory cytokines/chemokines, monocyte chemoattractant protein-1 (MCP-1) or Ccl2 has emerged as a key mediator of inflammation following tissue injury. Ccl2 is a member of the C-C chemokine family and signals at least in part through its receptor CCR2. Both Ccl2 and its receptor CCR2 are induced and are mechanistically linked to the pathogenesis of many chronic diseases, including neurodegenerative diseases^11,12^, inflammatory bowel disease^13^, rheumatoid arthritis^14^, inflammation-associated insulin resistance^15,16^, obesity, diabetes, cardiovascular diseases^17^, and atherosclerosis^18^.

Ccl2 has also been implicated as a key mediator of chronic kidney disease in animal models^19–22^ as well as in humans^23,24^. Pharmacological inhibition of Ccl2 has been shown to reduce chronic renal damage in lupus nephritis^25^, improve podocyte function in diabetic nephropathy^26^, and to improve renal function in diabetic patients with albuminuria^27^. Inhibition of the renin-angiotensin system reduces Ccl2 mediated inflammation in a variety of chronic injury models^6,28,29^.

Using the murine 2K1C model of RVH, we have previously shown that Ccl2 is induced within 24 hours of surgery to establish RAS, prior to the onset of interstitial inflammation, interstitial fibrosis, or tubular atrophy^20^. A CCR2 selective inhibitor protected the stenotic kidney from development of chronic renal damage in this model^20^. It is recognized that other ligands (such as MCP3/CCL7) signal through CCR2^30,31^. It is also recognized that pharmacological inhibition might have “off-label” effects that may not be due to specific CCR2 inhibition. Based on these considerations, we tested the hypothesis that Ccl2 deficiency alone can protect the stenotic kidney from the development of severe interstitial fibrosis, tubular atrophy, and interstitial inflammation in mice with homozygous deletion of the Ccl2 gene (Ccl2 KO mice) subjected to unilateral renal artery stenosis.

## Materials and Methods

### Animals

Male Ccl2 knockout (B6. 129S4-*Ccl2*^*tm1*Rol^/J) (KO) and wild type (C57BL6/J) (WT) mice were purchased from the Jackson Laboratory (Bar Harbor, ME). WT mice (N = 27 RAS, N = 9 sham) and Ccl2 KO (N = 24 RAS, N = 9 sham) mice underwent RAS or Sham surgery at 8–10 weeks of age. RAS surgery was done by placing a polytetrafluoroethylene cuff around the right renal artery using two sutures to secure the cuff in place as described previously^32^; sham surgeries were performed by isolating and manipulating the right renal artery without cuff placement. All of the animal procedures were approved by the Mayo Clinic Institutional Animal Care and Use Committee (IACUC) prior to conducting any experiment. These animal procedures were conducted in accordance with National Institutes of Health Guide for the Care and Use of Laboratory Animals.

### Imaging protocol and Analysis

MRI studies were performed on a subset of animals (N = 13 WT RAS, N = 4 WT Sham, N = 12 Ccl2 KO RAS, N = 4 Ccl2 KO Sham) employing a vertical 16.4T scanner equipped with a 38 mm inner diameter birdcage coil (Bruker Biospin, Billerica, MA). The images for the kidney volume determination were collected axially using respiratory gated 3D Fast Imaging with Steady Precession (3D-FISP) and reconstructed into 3D volumetric images. The kidney volume for cortex and medulla was then assessed from 3D-FISP using Analyze software (Biomedical Imaging Resource, Mayo Clinic, MN) and reported as sum of both cortex and medulla. Perfusion was assessed by arterial spin labelling (ASL) method by using a Flow-sensitive Alternating Inversion Recovery (FAIR) sequence with Rapid Acquisition with Relaxation Enhancement (RARE) as described earlier^33^. Perfusion of cortex and medulla in the stenotic kidney was calculated using the ASL tool module of Bruker-Paravision software and reported as the sum of both compartments. Blood flow for cortex and medulla was calculated by multiplying the perfusion times the volume of corresponding compartment and reported as their sum.

Renal oxygenation was assessed by BOLD-MRI imaging using a respiration-gated 3D multi-echo gradient echo sequence^33^. The imaging parameters used were: TR 200 ms; TE 3.5–24.5 ms; echo number 8; flip-angle 25°; slab thickness 1 mm; FOV 2.56 × 2.56 cm^2^; matrix size 128 × 128 × 8; number of averages 2.” 8 images were reconstructed after zero-filling the k-space data to 256 × 256 for BOLD MRI. T_2_* was quantified by pixel-wise mono-exponential fitting on the averaged magnitude of all 8 images over echo times. R_2_* (1/T_2_*), was used as an index of blood oxygenation level. ROIs were drawn on T_2_ images for BOLD analysis.

### Animal Blood Pressure Measurement

Non-invasive method using the tail cuff and volume pressure recording (VPR) sensor technology was used on conscious mice to record the blood pressure measurements at baseline and following surgery (CODA 6, Kent Scientific Corporation, Torrington, Connecticut, USA).

### Plasma Renin Activity

Plasma was available from 21 WT RAS, 7 WT Sham, 21 Ccl2 KO RAS, and 8 Ccl2 KO Sham mice. Blood was collected via the inferior vena cava four weeks after surgery; plasma was separated from it by centrifugation and stored at −80 °C until the time of assay. Quantitative determination of plasma renin content was done by radioimmunoassay as described earlier^20^.

### Renal Function

Renal function was assessed by the measurement of serum creatinine and blood urea nitrogen (BUN) levels at 24 h after ischemia using a Creatinine Analyzer 2 and a BUN Analyzer 2 (Beckman Instruments, Fullerton, CA) as previously described^34^.

### Histology and immunohistochemical analysis

Histologic and immunohistochemical analysis was performed on the subset of animals for which imaging studies were performed (see above). The kidney tissues were fixed in 10% neutral buffered formalin and processed using standard techniques. 5 µm histological sections were prepared and stained with hematoxylin-eosin (H&E). Immunohistochemical staining was done for anti-F4/80 (1:200, Abd Serotec Raleigh, NC) and anti-CD3 (1:100, Dako Agilent, Santa Clara, CA). Sections were stained with Sirius red to quantitate matrix deposition. All measurements and quantifications were performed in a blinded fashion using Olympus Bx50 microscope (Olympus Optical Co. Ltd., Buffalo Grove, IL, USA), Micropublisher 3.3 RTV camera (QImaging, Surrey, BC, Canada). The area of atrophy was calculated on H&E sections as percentage of atrophic tubules over the cortical area, as previously described^9,20^.

Quantitative analysis for F4/80 CD3, and Sirius red was done by calculating % positive stained area for F4/80, CD3, and Sirius red using NIS elements BR 4.13.00 64-bit image analysis system (Nikon Instruments INC., Melville, NY) at 200X magnification.

### Real-time PCR

Total RNA was extracted from stenotic kidney tissues using RNeasy plus Mini kit (Qiagen, Valencia, CA) (N = 17 WT RAS, N = 6 WT Sham, N = 20 Ccl2 KO RAS, N = 8 KO Sham). RNA quantification was done using spectrophotometry (NanoDrop Technologies, Wilmington, DE). RNA quality was assessed using Agilent 2100 Bioanalyzer (Agilent Technologies, Santa Clara, CA, USA). First-strand cDNA was prepared from total RNA using iScript cDNA synthesis kit (Bio-Rad, Hercules, CA). Real time PCR amplification reactions were performed on a Bio-Rad IQ5 real-time PCR detection system. The sequences for primers used in the studies were described earlier^20^ except *Gata3* (Forward:5′-AAG CTC AGT ATC CGC TGA CG-3′ Reverse: 5′-GAC ACC TCT GCA CCG TAG CC-3′) and *Rorc* (Forward: 5′-CCC TAG GCT TGC CTT GTA GG-3′ Reverse:5′-CCC ATC TAC CCC ACA GCT TC-3′). Commercially available primers for *Gapdh*, *Acta2* and *Col3A* were used (ThermoFisher Scientific, Waltham, MA).

### Statistical analysis

Data are presented as means ± SEM. ANOVA or *t-*test were performed for comparison between groups and post hoc Tukey/Dunn’s correction was used for multiple comparisons. *P* values < 0.05 were considered as significant. Statistical analyses were performed with GraphPad Prism 6 (GraphPad Software, La Jolla, CA).

### Data availability

All data generated or analyzed in this project are included within this paper.

## Results

### Blood pressure was elevated to a similar extent in Ccl2 KO RAS and WT RAS mice

**Figure 1:**
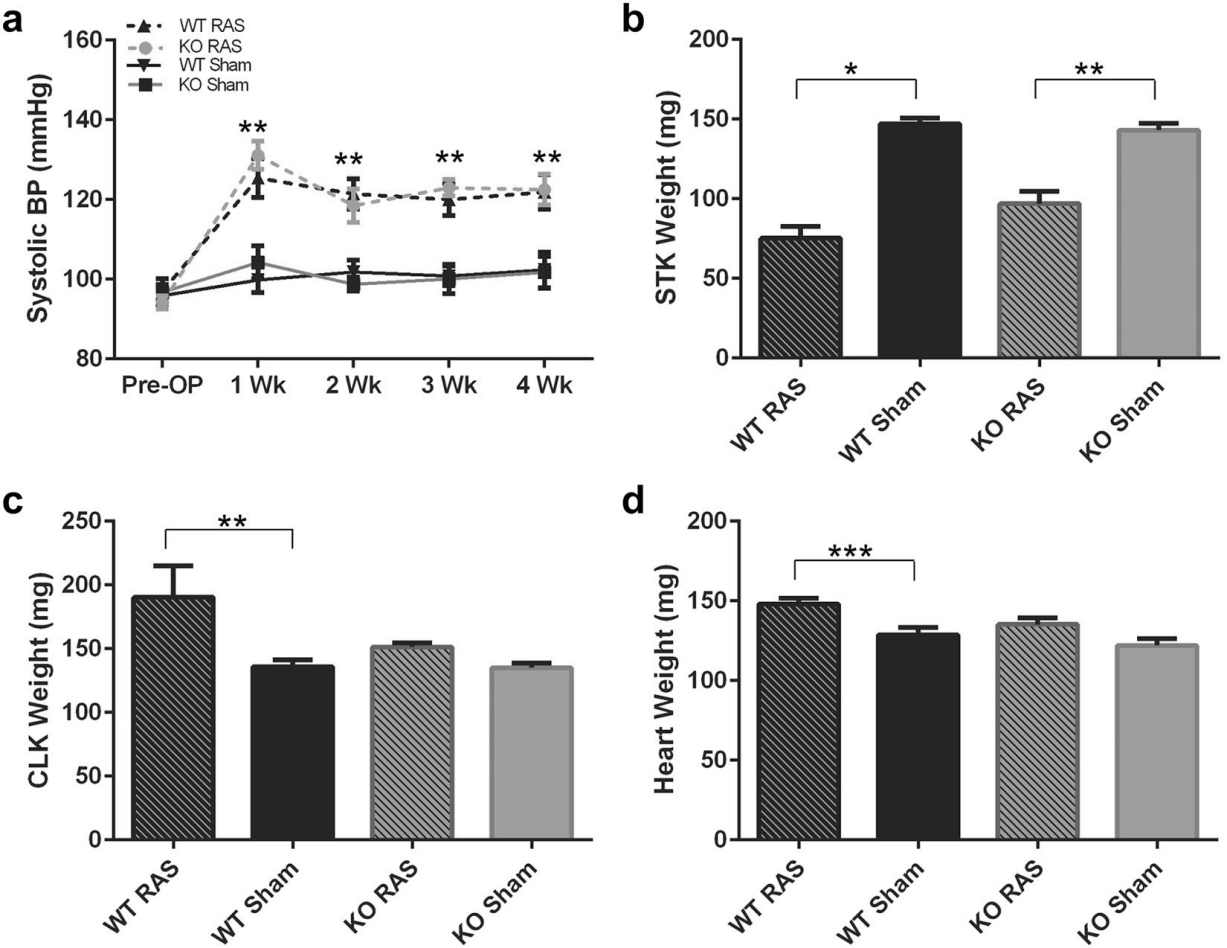
Blood Pressure, heart, and kidney weights of Ccl2 KO and WT mice. (**a**) Weekly blood pressure measurements in Ccl2 KO and WT mice. (**b**) Weight of the stenotic (cuffed) kidney, assessed at 4 weeks following surgery (STK = stenotic kidney). (**c**) Weight of the contralateral kidney, assessed at 4 weeks following surgery (CLK = contralateral kidney). (**d**) Heart weight, assessed at 4 weeks following surgery. *p < 0.0001, **p < 0.01, ***p < 0.05.

There were no differences in the total body weight of WT RAS, WT sham, Ccl2 KO RAS and Ccl2 sham mice. The mean systolic blood pressure in both WT and KO mice following surgery to establish unilateral renal artery stenosis was significantly higher compared to sham mice. However, there was no significant difference in blood pressure measured in WT RAS and Ccl2 KO RAS mice (Fig. 1a). As expected, the weight of the cuffed kidney was significantly reduced, compared to sham (Fig. 1b). In WT mice, the contralateral kidney became hypertrophic, as evidenced by a significant increase in weight (Fig. 1c). The heart weight was also increased in the WT RAS mice (Fig. 1d). In contrast, the contralateral kidney weights of the Ccl2 KO RAS mice was similar to that of Ccl2 sham mice (Fig. 1c) and the heart weights of Ccl2 KO RAS and Ccl2 KO sham mice were similar (Fig. 1d). There were no significant differences in plasma BUN, creatinine or plasma renin activity between any of the groups.

### Renal Blood flow was reduced to a similar extent in WT and Ccl2 KO RAS mice as assessed by MRI

**Figure 2:**
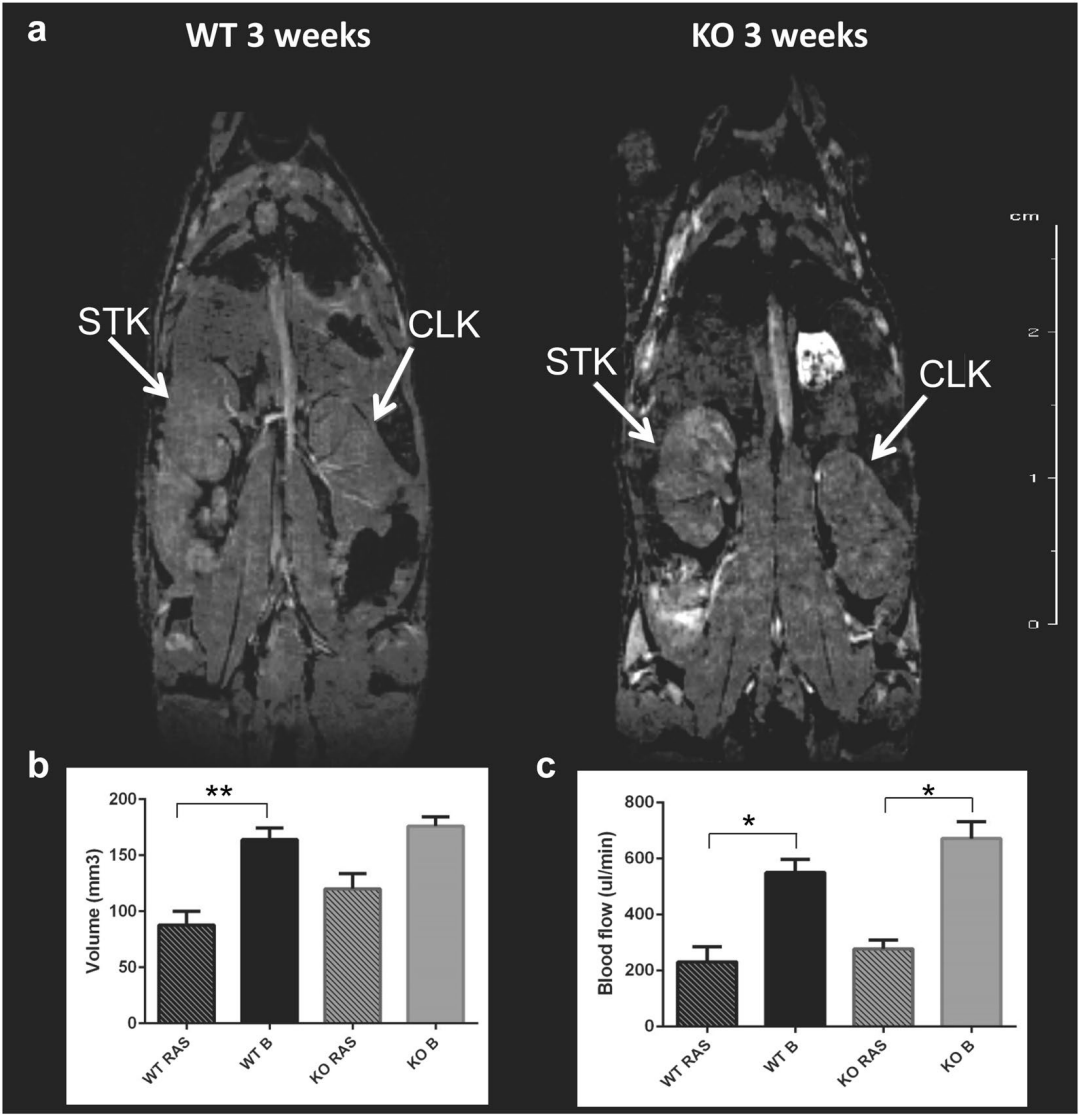
*In vivo* assessment of kidney size and blood flow by MRI 3 weeks following RAS surgery. (**a**) Coronal cross section MR images of WT and Ccl2 KO mice showing stenotic and contralateral kidney after RAS surgery. (**b**) Assessment of renal volume following RAS surgery. (**c**) Renal blood flow measured in the stenotic kidney. (‘B’ = baseline measurement assessed prior to surgery, ‘RAS’ = renal volume measurement at 3 weeks after surgery). *p ≤ 0.001, **p < 0.05.

**Figure 3:**
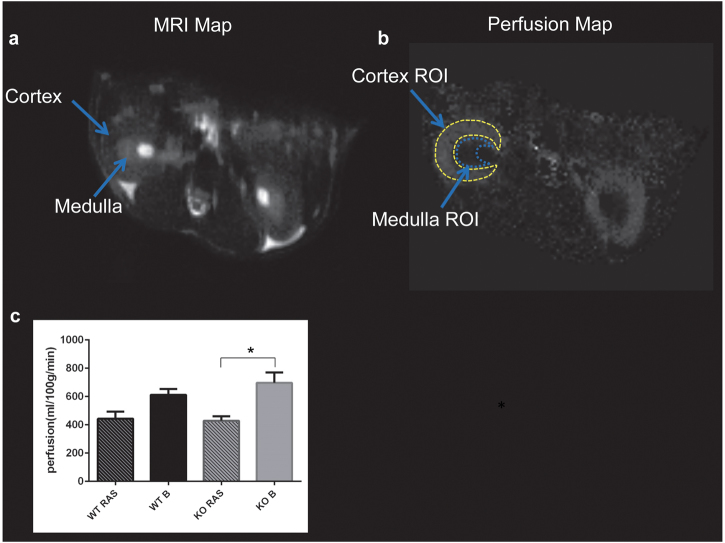
*In vivo* assessment of renal perfusion by MRI 3 weeks following RAS surgery. (**a**) Representative axial cross sectional MR image of the stenotic and contralateral kidney. (**b**) Representative perfusion map of renal cortex and medulla. (**c**) Assessment of renal perfusion at baseline and following RAS surgery. (‘B’ = baseline measurement prior to surgery, ‘RAS’ = renal perfusion measurement at 3 weeks after surgery). *p = 0.01.

**Figure 4:**
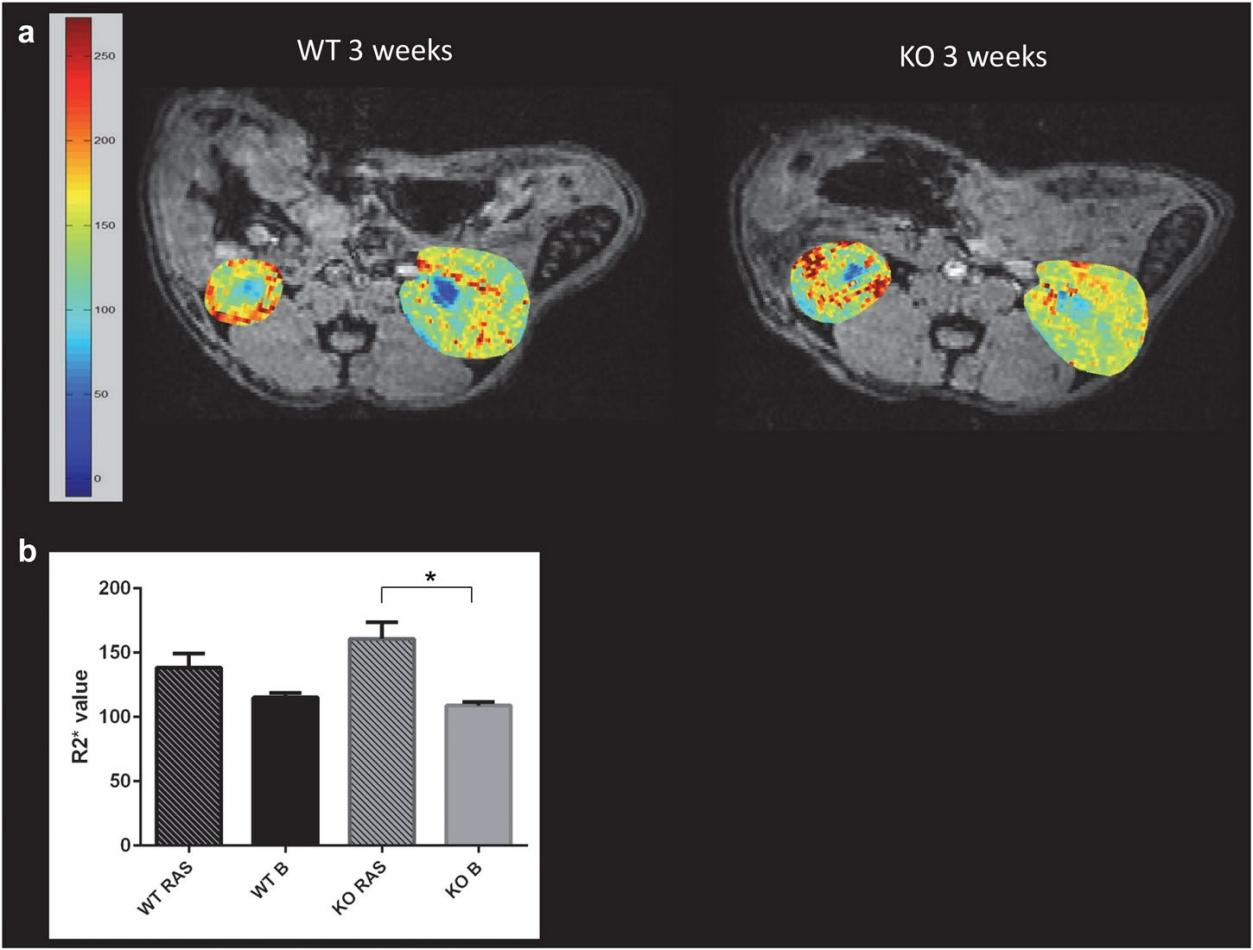
*In vivo* assessment of renal oxygenation by MR BOLD 3 weeks following RAS surgery. (**a**) Representative R_2_* maps obtained by blood oxygen dependent (BOLD) MRI for WT and Ccl2 KO mice following surgery. The increase in hypoxia is indicated by change in color intensity from yellow to red. (**b**) R_2_* value of renal cortex in STK in WT and KO mice at baseline and following surgery. (‘B’ = baseline measurement prior to surgery, ‘RAS’ = renal oxygenation measurement at 3 weeks after surgery). *p < 0.05.

The renal blood flow in both WT and Ccl2 KO RAS mice was reduced to similar extent following surgery to establish RVH (Fig. 2). The volume of the STK in WT RAS was significantly reduced compared to the pre-surgery baseline but no differences were observed between Ccl2 KO RAS and WT RAS mice at 3 weeks following surgery (1 week prior to the end of the experiment) (Fig. 2). Perfusion of the stenotic kidney in Ccl2 KO mice was significantly reduced, compared to its pre-surgery baseline (Fig. 3). There were no significant differences in perfusion of the stenotic kidneys of WT RAS or Ccl2 KO RAS mice. KO RAS mice showed significant elevation in cortical R_2_*_,_ which is a measure of hypoxia following RAS at 3 weeks compared to its baseline. Although the stenotic kidneys of WT RAS mice showed higher R_2_* values compared to their baseline, this difference did not reach significance (Fig. 4).

### The cuffed kidneys from Ccl2 KO RAS mice were protected from development of chronic renal damage

**Figure 5:**
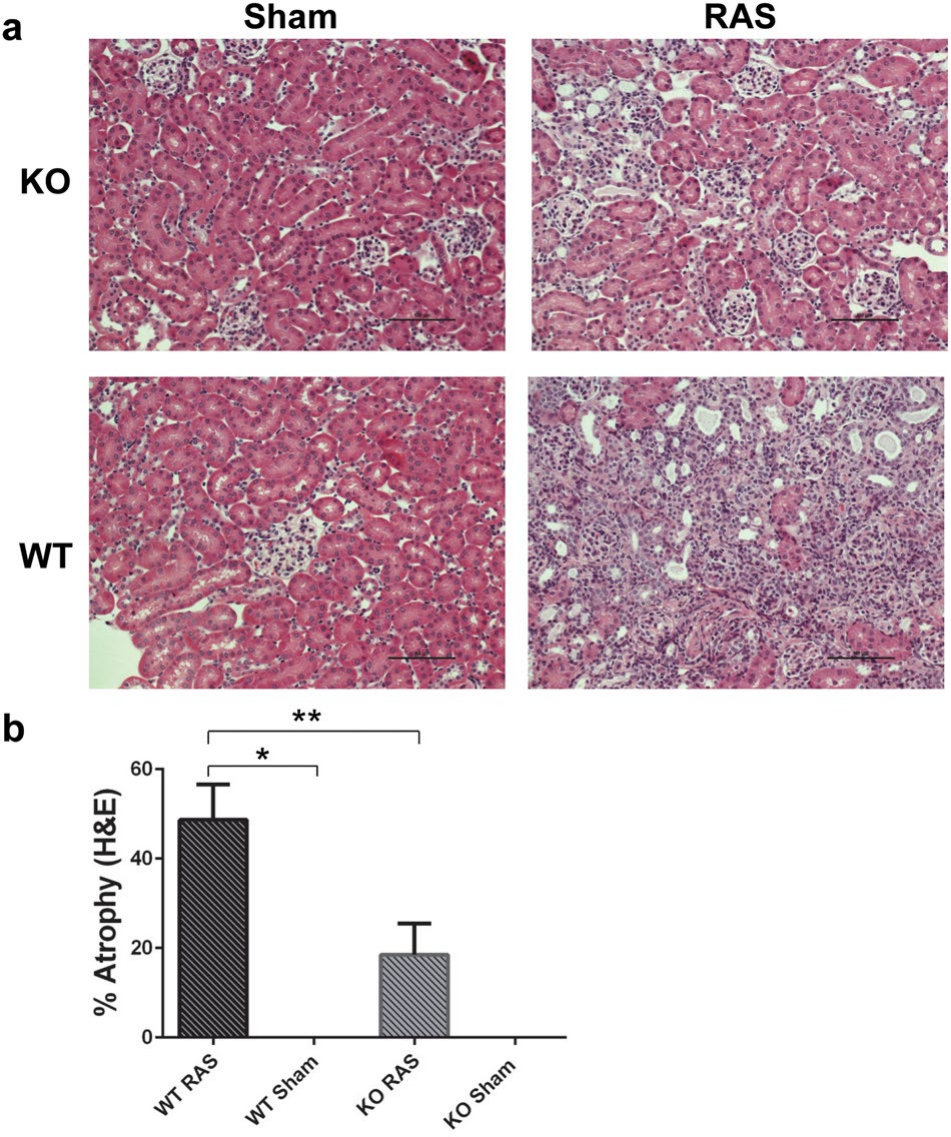
The stenotic kidney of Ccl2 KO mice is protected from development of chronic renal injury. (**a**) Representative histological images showing WT and KO following RAS/sham at 4 weeks following surgery (stained with H&E, 200X magnification). Scale bar represents 100 µm. (**b**) Semiquantitative assessment of tubular atrophy at 4 weeks following surgery. *p < 0.0001, **p < 0.05.

The stenotic kidney of KO mice showed protection against renal damage as assessed by “% atrophy cortical area” compared to WT mice (Fig. 5a,b) despite a similar elevation in blood pressure (Fig. 1a).

### The extent of interstitial fibrosis in Ccl2 KO RAS mice was less than that of WT RAS mice

**Figure 6:**
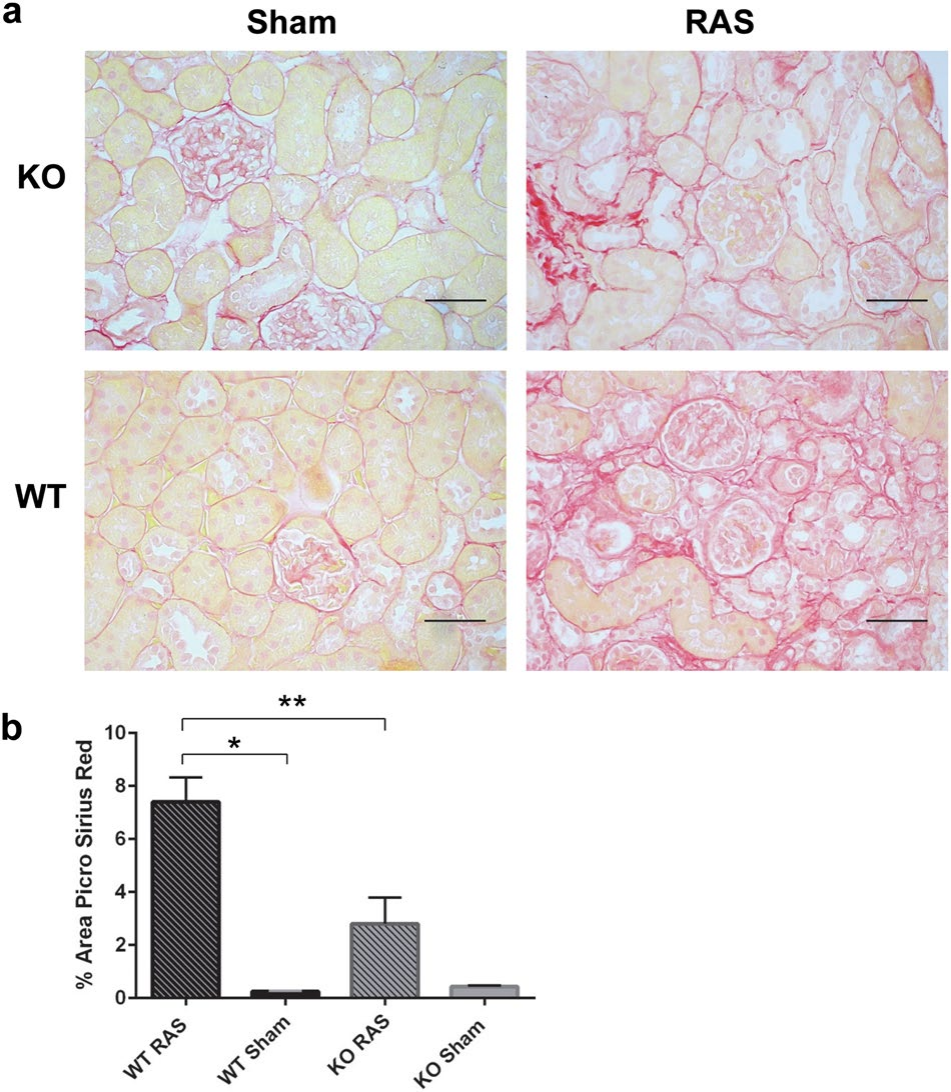
Collagen deposition, as assessed by Sirius red staining, is decreased in the stenotic kidney of Ccl2 KO RAS mice compared to WT RAS mice. (**a**) Representative images of renal cortex stained with Sirius red highlighting matrix deposition along tubular basement membranes and the interstitium (red staining). Scale bar represents 50 µm. (**b**) Quantitative analysis of the percent cortical surface area staining positively for extracellular matrix. *p ≤ 0.0001, **p < 0.01

Quantitative assessment of interstitial fibrosis was performed on Sirius red stained sections of renal cortex. The extent of interstitial fibrosis was significantly greater in WT RAS mice than in Ccl2 KO RAS mice (Fig. 6).

### Macrophage influx is decreased in Ccl2 KO mice compared to WT mice following RAS surgery

**Figure 7:**
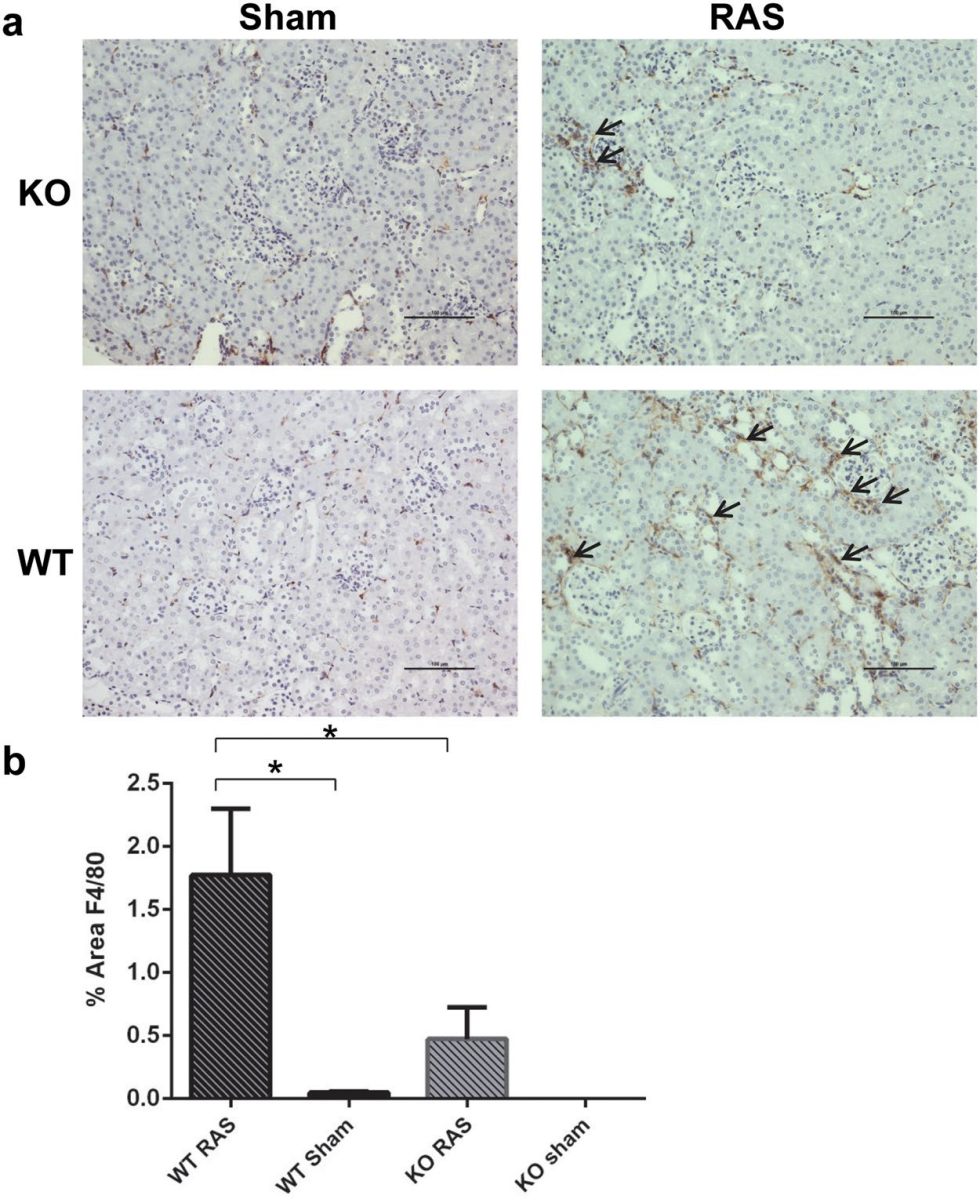
Macrophage influx, as assessed by F4/80 staining, is decreased in the stenotic kidney of Ccl2 KO RAS mice compared to WT RAS mice. (**a**) Immunohistochemical staining with anti-F4/80 antibody. The arrowheads highlight regions of positive staining for F4/80. Scale bar represents 100 µm. (**b**) Quantitative analysis of the percent of total cortical surface area staining positively for F4/80. *p < 0.05.

**Figure 8:**
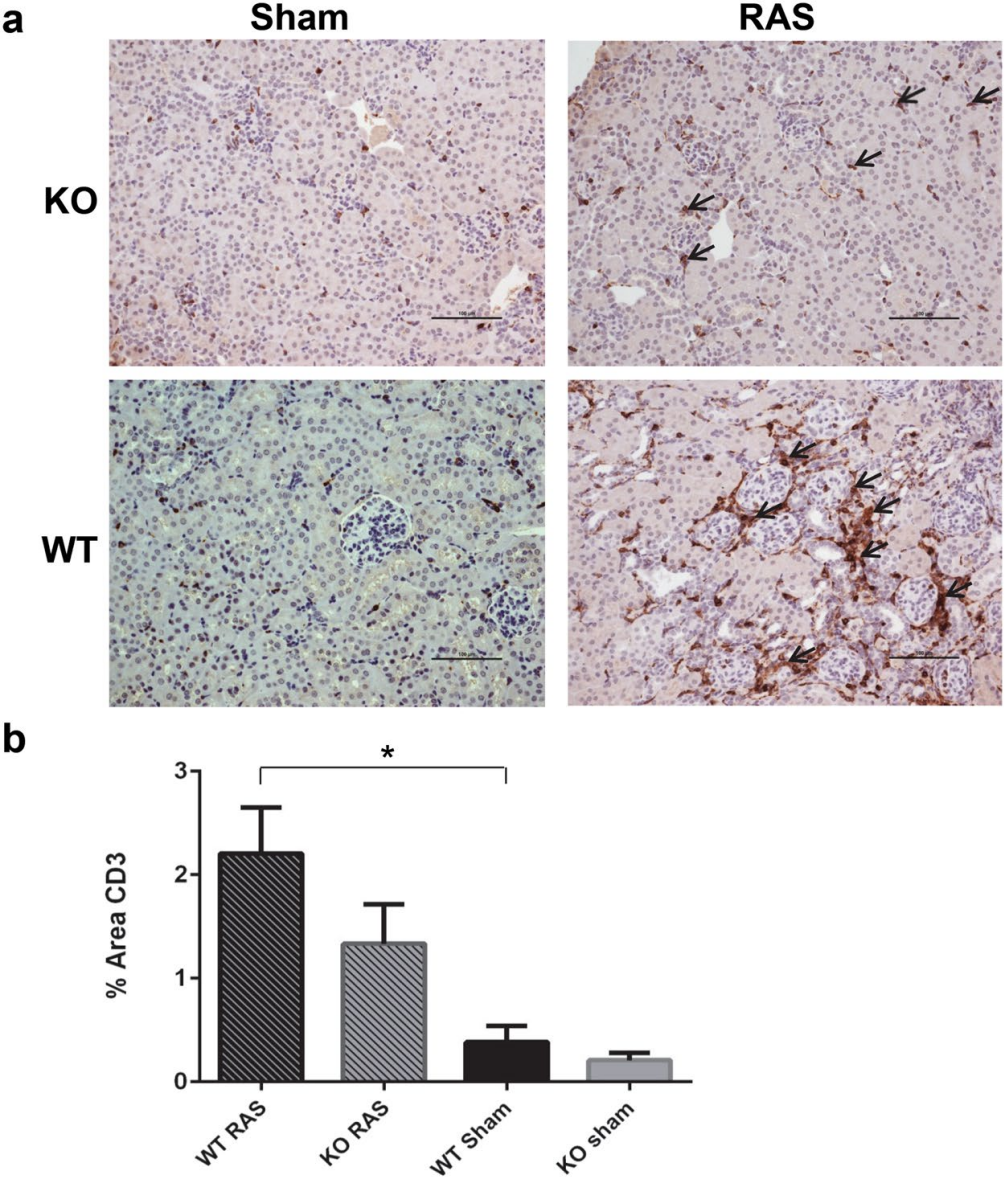
Lymphocyte influx, as assessed by immunohistochemical staining with anti CD3 antibody. (**a**) Representative histological images of WT and Ccl2 KO mice studied at 4 weeks following surgery. Sections are stained with anti CD3 antibody (200X magnification). The arrow highlights regions of positive staining for CD3. Scale bar represents 100 µm. (**b**) Quantitative analysis of the percent of total cortical surface staining positively for CD3. *p < 0.05.

Macrophage influx was lower in the stenotic kidney of Ccl2 KO mice than WT mice with RAS, as assessed by the percentage of cortical surface areas staining positively for the macrophage marker F4/80 (Fig. 7). We did not find any significant differences in CD3 + T cell influx between Ccl2 KO and WT mice with RAS (Fig. 8).

### Expression of pro-inflammatory chemokines is lower in Ccl2 KO than WT mice following RAS surgery

**Figure 9:**
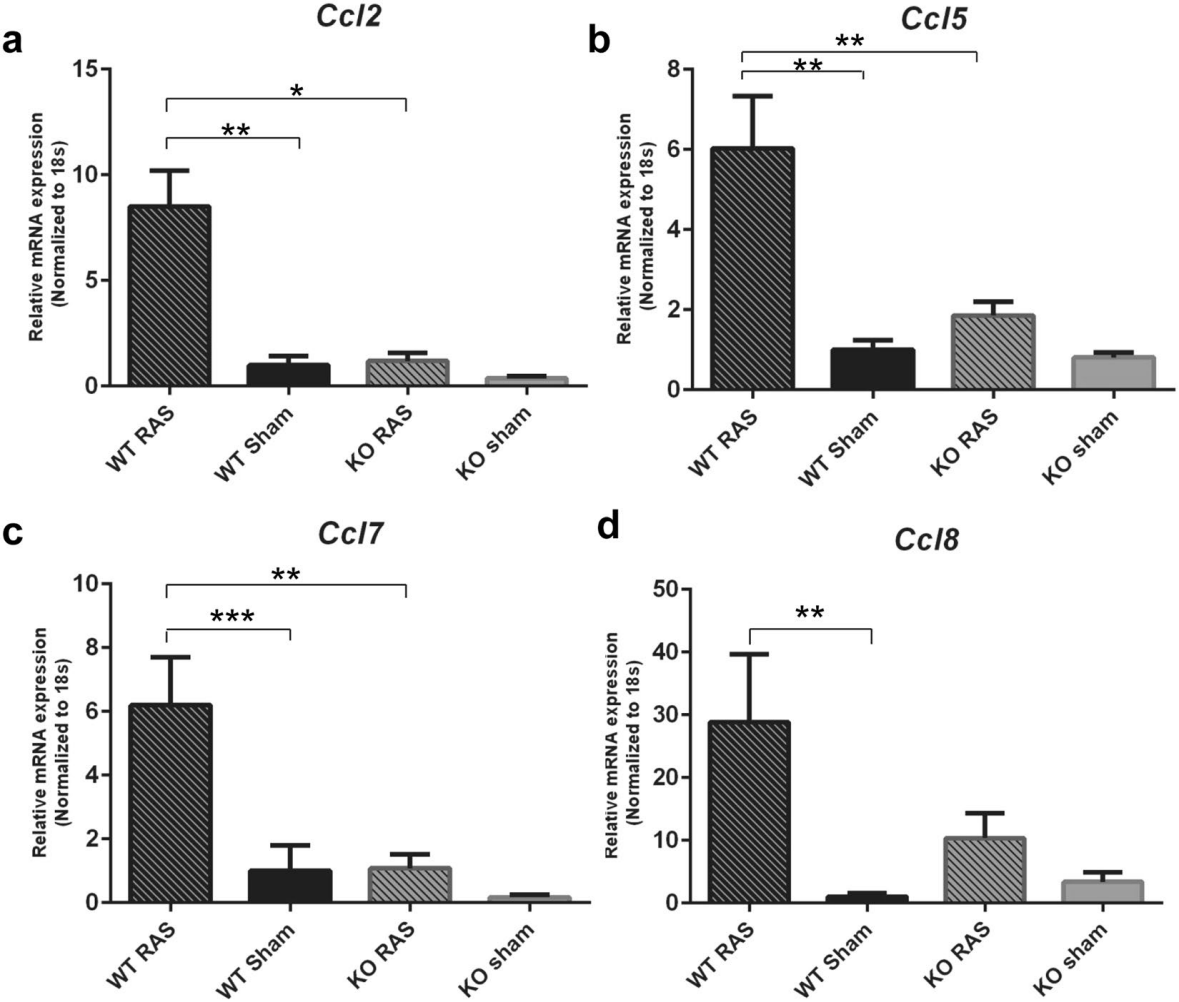
Pro-inflammatory chemokine expression in Ccl2 KO and WT RAS mice. Gene expression of (**a**) *Ccl2*, (**b**) *Ccl5*, **C)** *Ccl7*, (**d**) *Ccl8* in WT and KO mice. *p < 0.0001, **p < 0.01, ***p < 0.05.

*Ccl2* deficiency was confirmed using real time PCR in KO mice (Fig. 9a). *Ccl2* expression was significantly higher in WT RAS mice, compared to Ccl2 KO RAS and WT sham mice. Similar results were observed for the expression of *Ccl5*, *Ccl7 and Ccl8* (Fig. 9b,c,d respectively), although we did not observe any difference in *Ccl8* expression between WT and Ccl2 KO RAS.

**Figure 10:**
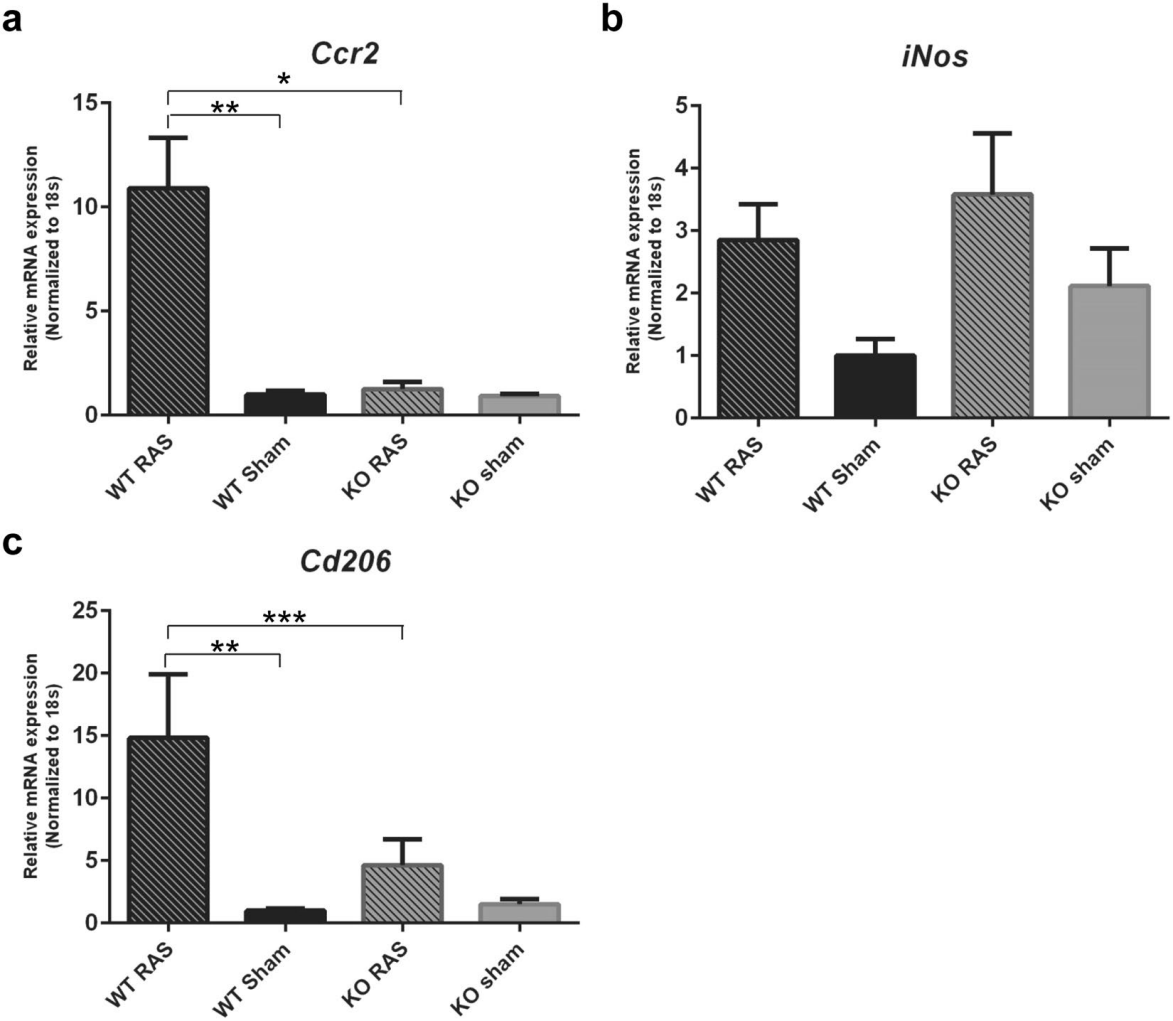
Pro-inflammatory chemokine expression in Ccl2 KO and WT RAS mice. The gene expression of (**a**) *Ccr2*, (**b**) *iNos*, (**c**) *Cd206* in WT and KO mice. *p < 0.0001, **p < 0.01, ***p < 0.05.

*Ccr2* expression was significantly higher in WT RAS mice, compared to Ccl2 KO RAS and WT sham mice (Fig. 10a). There were no significant differences in expression of *iNos* in any group (Fig. 10b). *Cd206* expression was higher in WT RAS mice, compared to WT sham and KO RAS mice (Fig. 10c).

**Figure 11:**
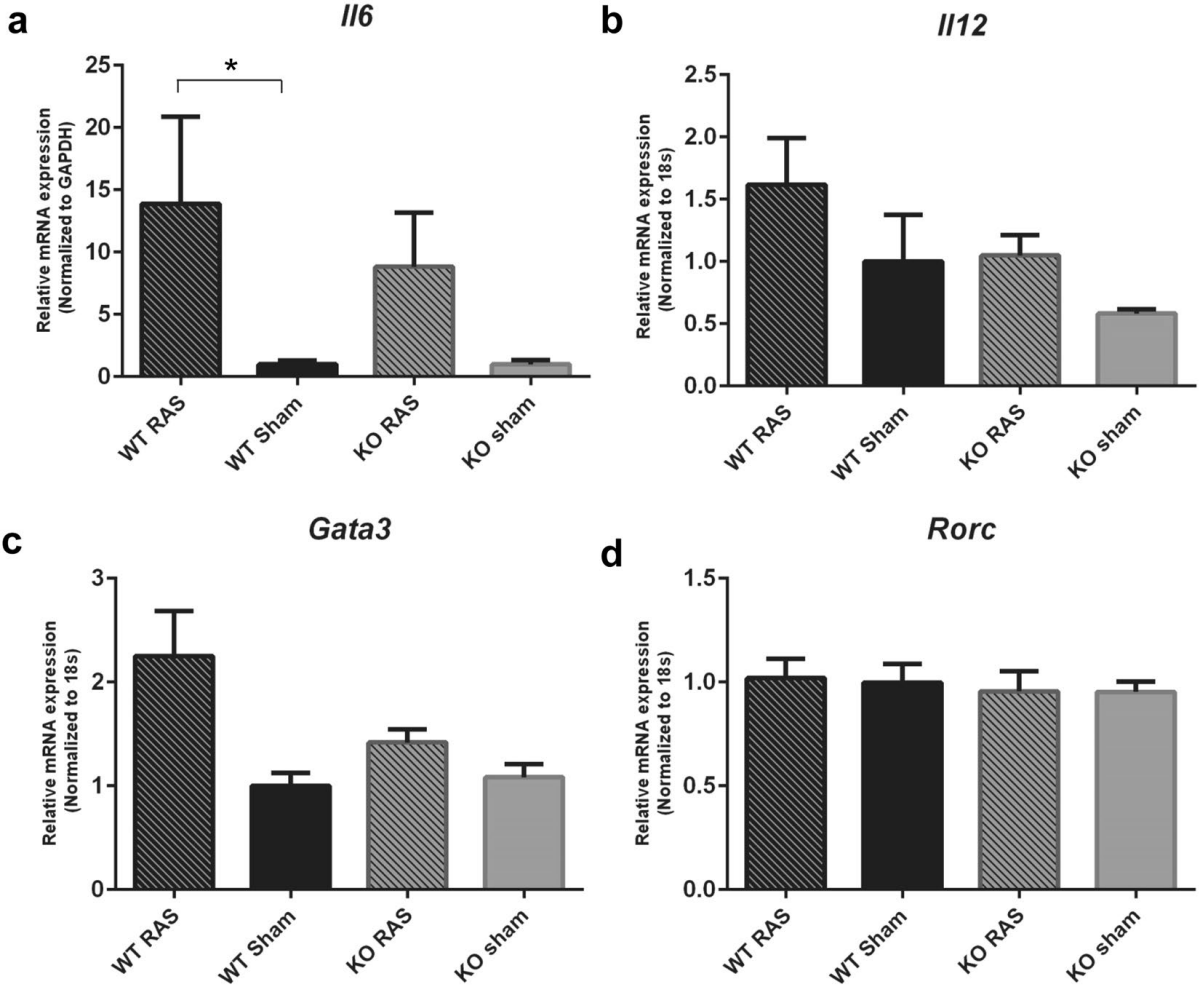
Pro-inflammatory chemokine expression in Ccl2 KO and WT RAS mice. The gene expression of (**a**) *Il6*, (**b**) *IL12*, (**c**) *Gata3*, (**d**) *Rorc* in WT and KO mice. *p < 0.05.

**Figure 12:**
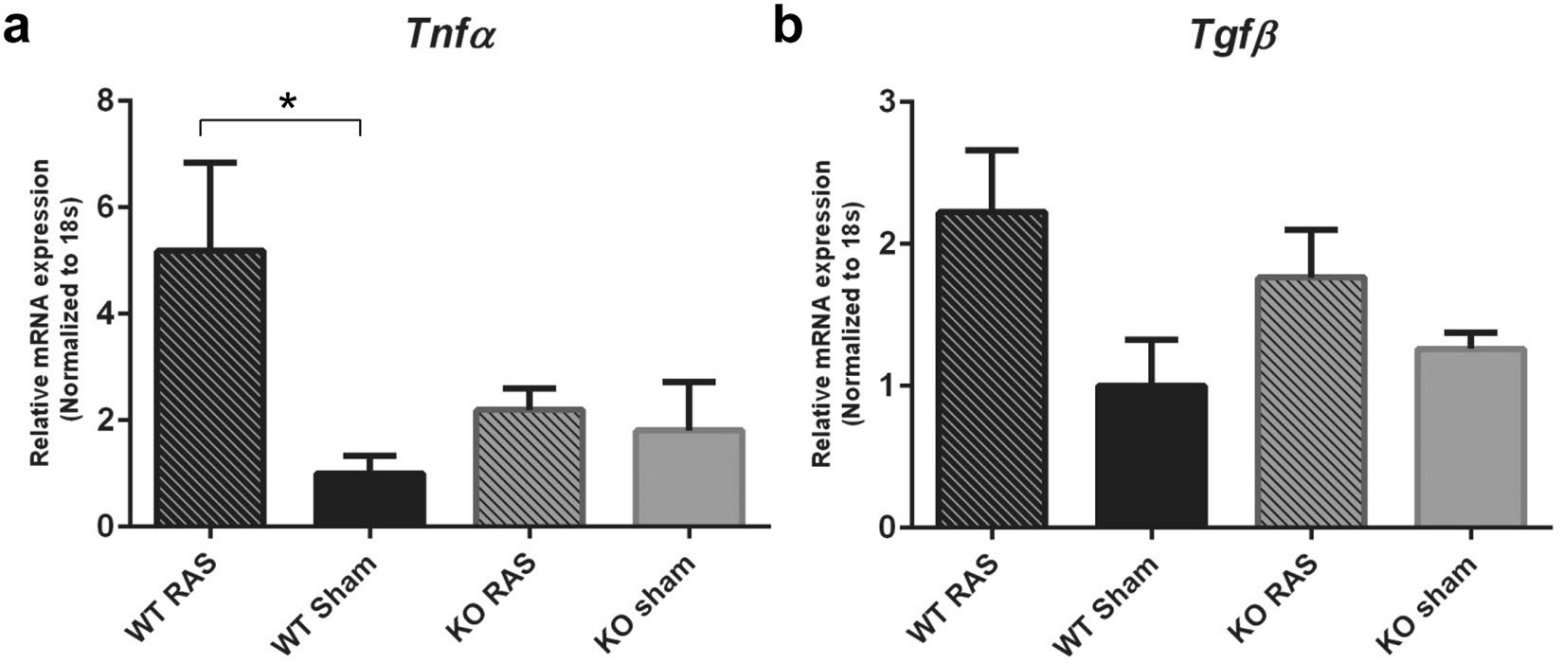
Pro-inflammatory chemokine expression in Ccl2 KO and WT RAS mice. The gene expression of (**a**) *TNFα*, (**b**) *Tgfβ*, in WT and KO mice. *p < 0.05.

*Il6* expression was significantly higher in WT RAS mice compared to WT sham (Fig. 11a). However, we did not find any differences in *Il12*, *Gata3*, *Rorc* expression in any of the groups (Fig. 11b,c,d respectively). Although, *TNFα* expression was significantly higher in WT RAS compared to sham (Fig. 12a), there were no differences in *TNFα* expression between WT and Ccl2 KO RAS. No difference in *Tgfβ* expression was observed in any group (Fig. 12b).

**Figure 13:**
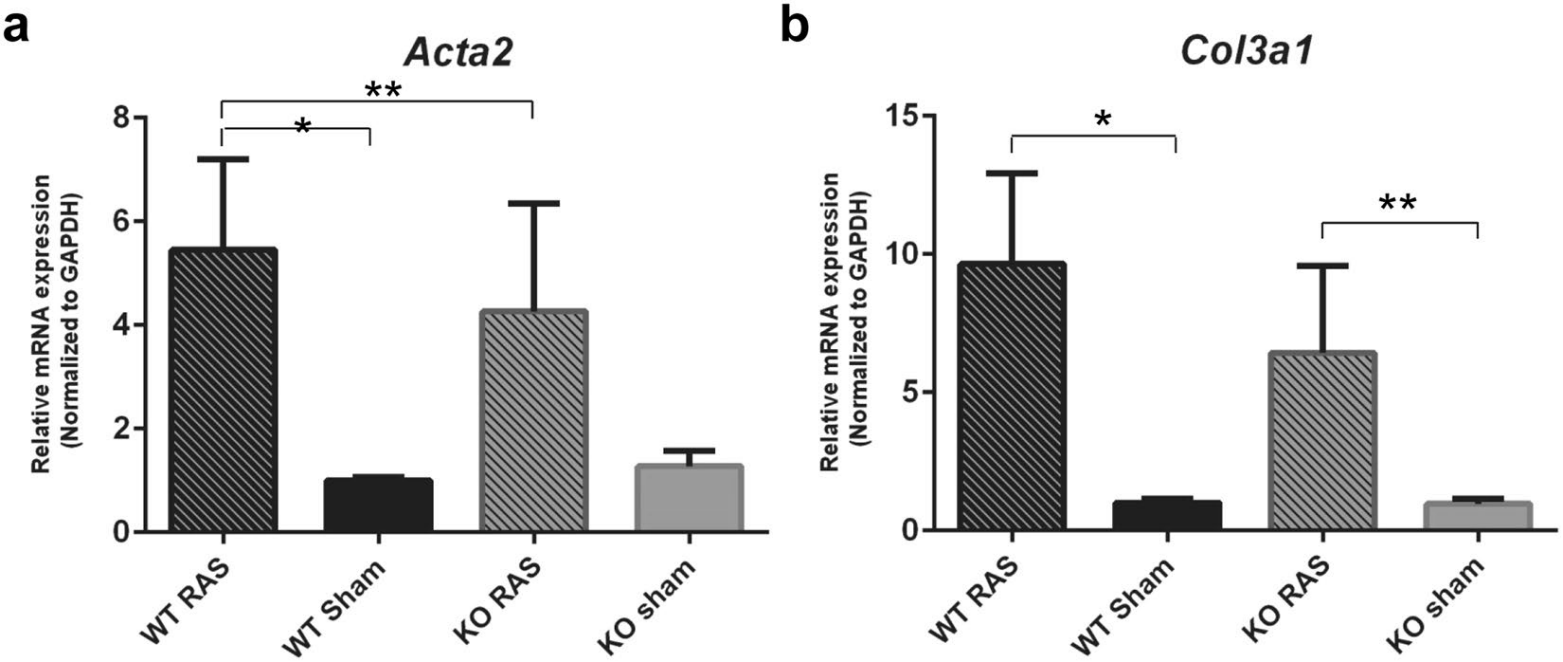
Alpha-actin and collagen 3 expression in Ccl2 KO and WT RAS mice. The gene expression of (**a**) *Acta2*, (**b**) *Col3A1* in WT and KO mice. *p < 0.01, **p < 0.05.

*Acta2* and *Col3A1* expression was significantly higher in WT RAS mice compared to its sham (Fig. 13a and b), whereas, *Col3A1* expression was significantly elevated in the KO RAS mice compared to the KO sham group (Fig. 13b). Acta2 expression was significantly higher in WT RAS mice than Ccl2 KO RAS mice (Fig. 13a). However, differences in *Col3A1* expression between Ccl2 KO and WT RAS mice did not reach statistical significance.

## Discussion

Our studies provide further evidence that Ccl2 plays a key role in mediating renal inflammation in RVH and its deficiency alone can protect against the chronic renal injury. Despite a similar flow reduction in the stenotic kidneys of WT and Ccl2 KO mice, the stenotic kidney of Ccl2 KO mice showed significant protection against renal injury, as evidenced by less cortical atrophy, compared to WT RAS. The R2* value, a measure of renal hypoxia, was significantly higher in the STK of KO mice which could be due to the fact that perfusion was significantly reduced in KO RAS mice, whereas perfusion was maintained in WT RAS mice, albeit at a reduced cortical volume. Plasma BUN and creatinine were within normal limits in all groups, indicating that the contralateral kidney was able to compensate for the chronic damage which developed in the stenotic kidney. These findings are consistent with observations that BUN and creatinine are relatively normal in patients who undergo unilateral nephrectomy.

Although, blood pressure was increased to a similar extent in KO and WT RAS mice, the heart weight was significantly higher than sham in WT RAS but not Ccl2 KO RAS mice. Similarly, the contralateral (non-cuffed) kidney showed a higher weight in WT RAS but not Ccl2 KO RAS mice. Reduction of chronic renal injury in the STK of KO RAS mice was associated with less macrophage, but not T cell infiltration.

In our previous study, we found that pharmacologic inhibition of CCR2 reduced *Ccl5* and *TNFα* expression, but not *Ccl2*, *Il*-*6*, *Il*-10, *Il*-12, or *Tgfβ* expression^20^. In the current study, we demonstrate that Ccl2 deficiency significantly reduces expression of other pro-inflammatory molecules, including *Ccl5*, *Ccl7*, *Ccl8*, *Ccr2*, and the M2 macrophage marker *Cd206*. Unlike the CCR2 inhibitor, *TNFα* expression was not significantly reduced in Ccl2 KO RAS mice, compared to WT RAS animals, raising the possibility that TNF expression may not directly involve Ccl2-CCR2 signaling.

The lack of cardiac hypertrophy in KO RAS mice contrasts with our studies of other signaling pathways culminating in renal atrophy in RAS. The STK of RAS mice treated with the p38 inhibitor SB203580 was protected from development of renal inflammation, tubular atrophy, and interstitial fibrosis. However, SB203580 had no effect on blood pressure, plasma renin activity, or heart weights compared to vehicle treated RAS mice^35^. Similarly, the STK of mice with deficiency of Smad3, a critical intermediate of TGF-β signaling, was protected from development of renal atrophy, interstitial fibrosis, and tubular atrophy. Although, blood pressure was increased to a similar extent in Smad3 KO and WT RAS mice at 3 days after surgery, blood pressure after that time point was significantly lower in Smad3 KO RAS mice than WT mice^32,36^. Despite this, the heart weight to body weight ratio was significantly higher in the Smad3 KO RAS mice than WT RAS mice. Furthermore, the Smad3 KO RAS mice had a severe cardiac phenotype characterized by myocardial inflammation with extensive myocyte necrosis, which was associated with sudden death in Smad3 KO RAS mice^36^. Based on these considerations, we propose that Ccl2 may provide a better therapeutic target to reduce chronic renal damage and to protect the heart than p38 or Smad3 inhibition.

Although, our studies support a central role for Ccl2 in reducing inflammatory responses to renal injury, there are several limitations. We demonstrated that mice with global Ccl2 deficiency are protected from development of renal atrophy in RVH; we have not established whether Ccl2 signaling in parenchymal cells or in bone marrow derived infiltrating inflammatory cells is responsible for this protective effect. Studies to address this issue will be important to determine whether more specific therapy should target immune/inflammatory cells or parenchymal cells to prevent development of chronic renal and cardiovascular disease in RVH. Not all studies support a protective role for Ccl2 deficiency in renal disease. In a renal ischemia-reperfusion model, Ccl2 deficiency exacerbated renal damage^37^. With that caveat in mind, human studies in patients with chronic renal disease show a strong correlation between urinary Ccl2 excretion, albuminuria, and renal infiltration of macrophages^23^. We conclude that therapy directed towards Ccl2 signaling may provide a novel therapeutic target to prevent progression renal and possibly cardiovascular disease in RVH.
